# Elevated IP-10 at the Protein and Gene Level Associates With Pulmonary TB

**DOI:** 10.3389/fcimb.2022.908144

**Published:** 2022-05-27

**Authors:** Kimone L. Fisher, Denelle Moodley, Kerishka Rajkumar-Bhugeloo, Omolara O. Baiyegunhi, Farina Karim, Hlumani Ndlovu, Thumbi Ndung’u, Mohlopheni J. Marakalala

**Affiliations:** ^1^ Africa Health Research Institute, Nelson R. Mandela School of Medicine, University KwaZulu-Natal, Durban, South Africa; ^2^ Human Immunodeficiency Virus (HIV) Pathogenesis Programme, Doris Duke Medical Research Institute, Nelson R. Mandela School of Medicine, University of KwaZulu-Natal, Durban, South Africa; ^3^ Department of Integrative Biomedical Sciences, Division of Chemical and Systems Biology, Faculty of Health Sciences, University of Cape Town, Cape Town, South Africa; ^4^ Division of Infection and Immunity, University College London, London, United Kingdom

**Keywords:** latent TB, tuberculosis, biomarkers, IP-10, TB diagnosis, IL-1RA, disease progression

## Abstract

There is an urgent need for accurate and sensitive diagnostic tools that can overcome the current challenge to distinguish individuals with latent tuberculosis infection (LTBI) from individuals with active tuberculosis (TB). Recent literature has suggested that a group of cytokines may serve as biomarkers of TB disease progression. Using a multiplex ELISA, we quantified 27 circulatory markers present within the unstimulated plasma of individuals in Durban, South Africa who were healthy (n=20), LTBI (n=13), or had active TB (n=30). RT-qPCR was performed to measure gene expression of the cytokines of interest, using RNA isolated from healthy (n=20), LTBI (n=20), or active TB (n=30). We found that at the protein level, IL-1RA, IL-6, and IP-10 were significantly more abundant in participants with active TB (p< 0.05) compared to those with LTBI individuals. IP-10 also showed the strongest association with active TB compared to healthy and LTBI at mRNA level. Our data shows that these proteins may serve as biomarkers of TB at both the protein and gene level.

## Introduction

*Mycobacterium tuberculosis* (Mtb) remains the leading cause of death by a single infectious agent (WHO, 2021). According to the World Health Organization (WHO) ~ 1.5 million people died from tuberculosis (TB) in 2021 (WHO, 2021). TB ranges from latent, to subclinical disease, and active TB disease (Pai et al., 2016). However, diagnosing and differentiating between disease states is difficult, and diagnostic tools lack sensitivity (Dorman, 2015) and can result in improper treatment or misdiagnosis.

Current methods of identifying those with TB infection include smear microscopy by staining for acid fast bacilli and by culturing Mtb, which is a tedious and a time-consuming process [as reviewed by (Sudbury et al., 2020)]. In addition, recent diagnostic tools such as GeneXpert are expensive and are mainly located at provincial hospitals and not in rural clinics, emphasizing the need for affordable and accessible means of diagnosing TB (Bassett et al., 2019). Current gold standards of testing for LTBI is the tuberculin skin test (TST) and the interferon gamma release assay (IGRA), which confirms the presence of interferon (IFN)-γ production as a surrogate marker of T cell responses (Diel et al., 2011). However, TST and IGRA cannot distinguish between those with LTBI and TB, which is an important distinction to make when determining correct courses of treatment. Therefore, identifying potential biomarkers of protective immunity or surrogates of disease progression remains an important need in the fight against TB (Leem et al., 2018).

Several studies have investigated the validity of using Mtb-specific cytokines to discriminate between LTBI and TB (Sudbury et al., 2020). Various cytokines have been implicated in protection during TB disease, including IFN-γ and interleukin (IL)-1RA (Cooper, 2009; O'Garra et al., 2013; Moreira-Teixeira et al., 2018; Sia and Rengarajan, 2019). IFN-γ is a well-known immune correlate in TB (Reichler et al., 2020). Recently, a type 1 IFN inflammatory gene signature in the blood of mice was shown to exacerbate TB pathogenesis and drive lung pathological damage in mice, and also served to differentiate those with active TB (Moreira-Teixeira et al., 2020). However, studies have also suggested that it is difficult to identify a single cytokine as a biomarker of disease, suggesting that a group or several cytokines may be key in identifying potential bio-signatures of disease as well as immune correlates of TB (Weiner et al., 2013). Another study showed that IFN-γ, IL-2, IL-5 and IL-10, IL-1RA, and MCP-1 distinguished between individuals with LTBI and TB (Suzukawa et al., 2016). In addition, stimulating plasma with Mtb specific antigens is limited by its ability to only distinguish those who are infected with Mtb and those who have been BCG vaccinated (Richeldi, 2006; Parida and Kaufmann, 2010). Stimulating plasma does not allow for the discrimination of individuals who are LTBI and those with active TB, which poses a considerable challenge to accurately diagnosing TB (Yao et al., 2017). Identifying circulatory markers that can accurately and sensitively differentiate between LTBI and TB is important in narrowing the search for an appropriate diagnostic marker that can overcome the challenges associated with current diagnostic tools.

In this study, we assessed the validity of using plasma isolated from individuals diagnosed with TB using GeneXpert and those with LTBI, diagnosed using the IGRA. We report that in unstimulated plasma, the cytokines IL-1RA, IL-6, and the chemokine IP-10 discriminate between healthy, LTBI, and TB patients.

## Methods

### Study Design and Setting

This study included participants from healthcare facilities that service the eThekwini district in KwaZulu-Natal (KZN), South Africa. Participants were recruited from Kwadabeka clinic and

Prince Cyril Zulu Communicable Disease Centre. South Africa had a TB prevalence rate of 737 in 100,000 in 2017 (Richeldi, 2006) with eThekwini described as having the highest TB prevalence rate in KZN. This study was approved by the Biomedical Research Ethics Committee (BREC) at the University of KwaZulu-Natal (BE022/13). We confirm that all research was performed in accordance with relevant guidelines/regulations. Informed consent was obtained from all participants and/or their legal guardians. We recruited TB patients who were newly diagnosed as GeneXpert positive (n=30), and LTBI (n=20) who were quantiferon (QFT) positive and healthy individuals (n=20) (QFT negative). These participants were treatment naïve. Clinical characteristics of all 70 participants used in this study are listed in **Table 1**.

**Table 1:** Clinical characteristics of n=70 participants used in this study.

Characteristics	Healthy (IGRA-)	LTBI (IGRA+)	TB (GeneXpert+)
n	20	20	30
**Age (mean and SD)**	30.90± 12.59	32.00 ± 11.95	35.37 ± 10.93
**Sex**			
Male	35.% (7)	45% (9)	73.33% (22)
Female	65% (13)	55% (11)	23.33% (7)
Not disclosed			3.33% (1)
**Assays**			
Plasma- Bio-Plex	20	13	30
qPCR on whole blood RNA	20	20	30

### Sample Collection

Whole blood was collected in heparin tubes. There was 20 ml of blood layered onto Ficoll density gradient medium and centrifuged at 800×g for 30 min with breaks off. PBMCs were collected and stored in liquid nitrogen storage facilities based at the AHRI, Durban, South Africa. The plasma was collected by centrifuging whole blood at 1000 ×g for 10 min with acceleration at maximum and deceleration off. The plasma was collected and stored at -80°C until use. GeneXpert diagnostic experiments were conducted at the National Health Laboratory Services, Durban, South Africa, according to the manufacturer’s instructions (Cepheid, CA). QFT-gold plus (Qiagen) experiments were repeated by the AHRI diagnostic department according to the manufacturer’s instructions.

### Measurement of Plasma Cytokines by Multiplex ELISA

A multiplex Luminex assay was performed using the Bio-Plex 27 human cytokine screening panel, a 27-plex kit from Bio-Rad (Hercules, CA) to measure interleukin (IL)-1β, IL-1 receptor antagonist (IL-1RA), IL-2, IL-4, IL-5, IL-6, IL-7, IL-8, IL-9, IL-10, IL-12, IL-13, IL-15, IL-17, Eotaxin, fibroblast growth factor (FGF) basic, granulocyte colony stimulating factor (G-CSF), granulocyte macrophage colony stimulating factor (GM-CSF), interferon-γ (IFN- γ), interferon gamma inducible protein (IP-10), monocyte chemoattractant protein-1 (MCP-1), macrophage inflammatory protein alpha (MIP-1α), platelet derived growth factor (PDGF-bb), MIP-1β, regulated on activation, normal T cell expressed and secreted (RANTES), tumour necrosis factor (TNF- α), and vascular endothelial growth factor (VEGF). Assays were performed as per the manufacturer’s instructions and were obtained with the Bio-Plex 200 plate reader (CA). The sensitivity of the kit was 0.2-45.6 pg/ml for each of the 27-cytokine concentrations measured. The Bio-Plex-manager software version 6 was used to collect the data and a 5PL regression formula was used to generate the standard curves for each cytokine to interpolate the concentration of cytokines in the samples. Cytokines that were expressed lower than the lower limit of detection were reported as zero. The samples were diluted according to the manufacturer’s instruction for plasma samples.

### RNA Isolation and cDNA Synthesis

There was 10 ml of whole blood collected in PAXgene™ tubes. RNA was isolated from whole blood according to the manufacturer’s instructions using the Paxgene™ kit (PreAnalytix, Switzerland). Complimentary DNA (cDNA) was synthesized from isolated RNA using the protocol described by Bio-Rad reverse transcription kit (Bio-Rad, Hercules, CA). Briefly, RNA was adjusted to a concentration of 500 ng for cDNA synthesis. Appropriate 5-20 µl of nuclease free water, 1 µl of RNA, and 4 µl of iScript 5× reaction mix were added for each sample to a total reaction volume of 20 µl. The T100 thermocycler from Bio-Rad (Hercules, CA) was set to 5 min at 25°C, 30 min at 42°C, and 5 min at 85°C.

### Real Time Quantitative Polymerase Chain Reaction for Gene Expression Analysis

Real time quantitative polymerase chain reaction (RT-qPCR) was done on the candidate genes as per the manufacturer’s instruction. The total reaction volume was 10 µl. There was 1 µl of cDNA added to each well with 9 µl of mastermix consisting of the appropriate forward and reverse primer at 0.5 µl each, 5 µl of iTaq™ Universal SYBR green supermix (Bio-Rad, Hercules, CA), and 3 µl of nuclease free water. The CFX 96 thermocycler (Bio-Rad, Hercules, CA) was set to the following protocol: 30 sec at 95°C, 5 sec at 95°C, and 30 sec at 56°C for 39 cycles. The melt curve analysis was done at 65-95°C at 0.5°C increments.

### Primer Design

Primers were designed using the IDT primer design tool, PrimerQuest Tool, and sequences were blasted using the BlastN tool on NCBI. Primers were designed for IL-1RA, IL-6, and IP-10 (CXCL10) (see **Table 2**).

**Table 2:** Primers used for gene expression studies.

Target	Accession number	Forward primer	Reverse primer
IL-1RA	NM_001318914.2	5’-GCC TTC AGA ATC TGG GAT GTT-3’	5’-CGC CTT CGT CAG GCA TAT T-3’
IL-6	NM_000600	5’-TCT GGA TTC AAT GAG GAG ACT TG-3’	5’-GGA CTG CAG GAA CTC CTT AAA-3’
IP-10 (CXCL10)	NM_001565.4	5’- CTC TAA GTG GCA TTC AAG GAG TA-3’	5’-ACC CTT GGA AGA TGG GAA AG-3’

### Statistical Analysis

To identify any potential cytokine profiles that were associated with disease state, the data were log transformed and normalized and a principal component analysis (PCA) of the data was done. One-way ANOVA was done to compare disease groups, followed by a multiple comparison post-test using Tukey’s test. Values of *p*<0.05 were considered statistically significant. Data was analyzed using the GraphPad Prism version 9.4 software.

## Results

### Circulatory Cytokines May Distinguish Between Healthy, LTBI and Active TB Patients

**Figure 1:**
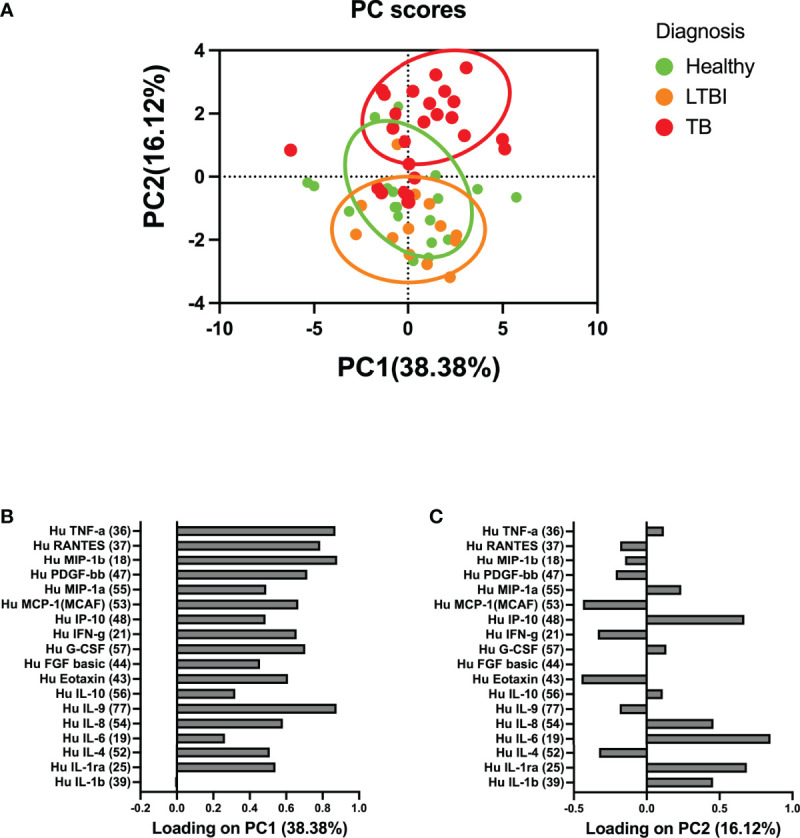
Principal component analysis (PCA) of the association between circulatory cytokines and TB disease states. **(A)** Circulatory cytokines separate according to whether individuals are healthy, latent TB infected, or with active TB disease. **(B)** PC1 accounted for 38.38% of the variance observed in the data set. **(C)** PC2 accounted for 16.12% of the variance observed in the data set. PC2 was used to define the association between circulatory cytokine and whether individuals were healthy, LTBI, or those who had active TB. Each dot represents a participant score on the loading components.

To determine if circulatory cytokines are associated with active TB and LTBI, we performed a multivariate principal component analysis using unsupervised clustering. Cytokine protein concentrations that segregated according to disease, along PC2 (16.12%), were associated with higher protein abundance of cytokines such as IL-1RA, IL-6, IL-8, and IP-10 (**Figures 1A, C**). The remainder of the cytokines were loaded on PC1 (38.38%, **Figures 1A, B**). Given the current understanding that a group of cytokines may serve as biomarkers of disease progression, these data suggest that certain cytokines may be differentially expressed in specific stages of disease.

### IL-1RA, IL-6, and IP-10 Are Abundant in TB Patients Compared to Healthy and LTBI

**Figure 2:**
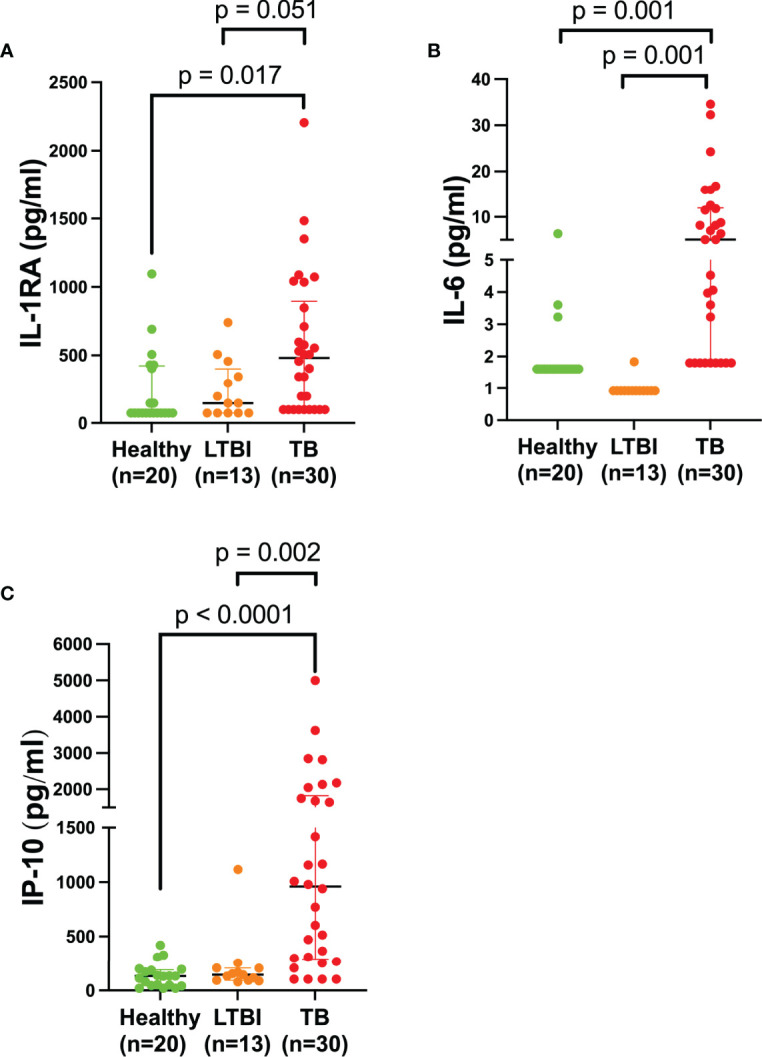
Plasma IL-RA, IL-6, and IP-10 are elevated in TB patients. Quantification of various cytokines in the plasma of healthy (n=20) individuals compared to LTBI (n=13) and TB (n=30) patients. Each dot represents a participant sample. **(A)** IL-1RA, **(B)** IL-6, and **(C)** IP-10 were quantified and measured in pg/ml. Significant differences were identified as those values that were less than *p*<0.05. Data was analyzed using the one-way ANOVA and followed by a multiple comparisons test using Tukey’s test. Tukey’s *p*-values have been reported in the above figure and illustrate the differences observed between groups.

Following the findings that some circulatory markers were associated with disease and that participant scores loaded more highly on PC2, we sought to investigate whether there were any significant inflammatory circulatory biomarkers that could distinguish healthy individuals, LTBI, and active TB patients. We found that there was a difference between at least one of the groups for IL-1RA (*p* = 0.009), IL-6 (*p* = 0.0001), and IP-10 (*p* < 0.0001). Tukey’s test found that IL-1RA (*p* < 0.017, 91% CI (-605.9 to -50.41), **Figure 2A**), IL-6 (*p* = 0.001, 91% CI (-10.91 to -2.357), **Figure 2B**), and IP-10 (*p* < 0.0001, 91% CI (-1667 to -511.3), **Figure 2C**) were abundant in the TB group, compared to the healthy group. IL-1RA (*p* = 0.051, 91% CI (-638.2 to 0.745), **Figure 2A**), IL-6 (*p* = 0.001, 91% CI (-12.59 to -2.755), **Figure 2B**), and IP-10 (*p* = 0.002, 91% CI (-1674 to -344.5), **Figure 2C**) were also more abundant in the TB group compared to the LTBI group. Protein abundance of all 27 cytokines analyzed are listed in **Table 3**.

**Table 3:** Absolute protein abundance of all cytokines assessed in this study.

Cytokine	Healthy		LTBI		TB	
	Median	IQR	Median	IQR	Median	IQR
IL-1β	1.165	1.165-1.175	0.205	0.205-0.205	1.08	1.08-1.08
IL-1RA	73.02	73.02-423.4	146	73.02-400.1	481.8	97.98-895.2
IL-2	N/A	N/A	N/A	N/A	N/A	N/A
IL-4	0.48	0.48-0.84	0.41	0.41-0.82	0.41	0.41-0.41
IL-5	N/A	N/A	N/A	N/A	N/A	N/A
IL-6	1.615	1.615-1.615	0.92	0.92-0.92	5.08	1.80-11.98
IL-7	N/A	N/A	N/A	N/A	N/A	N/A
IL-8	2.598	1.925-12.65	7.42	2.818-11.66	6.225	1.635-14.94
IL-9	207	124.9-328.9	228.2	168.5-311.4	181.9	110.3-274.9-
IL-10	1.295	1.295-2.266	2.590	1.295-3.510	1.295	1.295-2.59
IL-12 (p70)	N/A	N/A	N/A	N/A	N/A	N/A
IL-13	N/A	N/A	N/A	N/A	N/A	N/A
IL-15	N/A	N/A	N/A	N/A	N/A	N/A
IL-17	N/A	N/A	N/A	N/A	N/A	N/A
Eotaxin	8.035	2.533-29.30	6.870	4.425-30.06	5.370	3.990-17.66
FGF basic	8.245	8.245-8.245	8.245	8.245-12.37	9.885	9.885-9.885
G-CSF	62.40	33.20-116.1	94.04	71.27-149.7.0	113.6	45.10-156.5
GM-CSF	N/A	N/A	N/A	N/A	N/A	N/A
IFN- γ	1.855	1.350-7.620	3.710	2.360-5.07-	1.180	1.180-4.390
IP-10	131.9	46.84-192.4	145.8	93.98-207	958.7	291.3-1823
MCP-1	14.61	7.560-36.30	17.90	10.51-37.60	11.56	5.918-20.57
MIP-1 α	1.990	1.990-1.990	1.105	1.105-2.7	1.660	0.83-4.123
PDGF-bb	139.9	38.80-374.20	213.9	98.61-509.6	143.4	31.65-283.1
MIP-1 β	132.5	75.38-165.9	157.8	120.8-208.5	119.7	76.33-142.4
RANTES	3736	1441-6231	34.74	17.14-45.95	3034	1496-4326
TNF- α	36.12	18.42-42.24	34.74	17.14-45.95	40.89	24.30-53.97
VEGF	N/A	N/A	N/A	N/A	N/A	N/A

N/A, these values were lower than the limit of detection.

### Gene Expression of IP-10 Distinguishes Between Healthy, LTBI, and TB individuals

**Figure 3:**
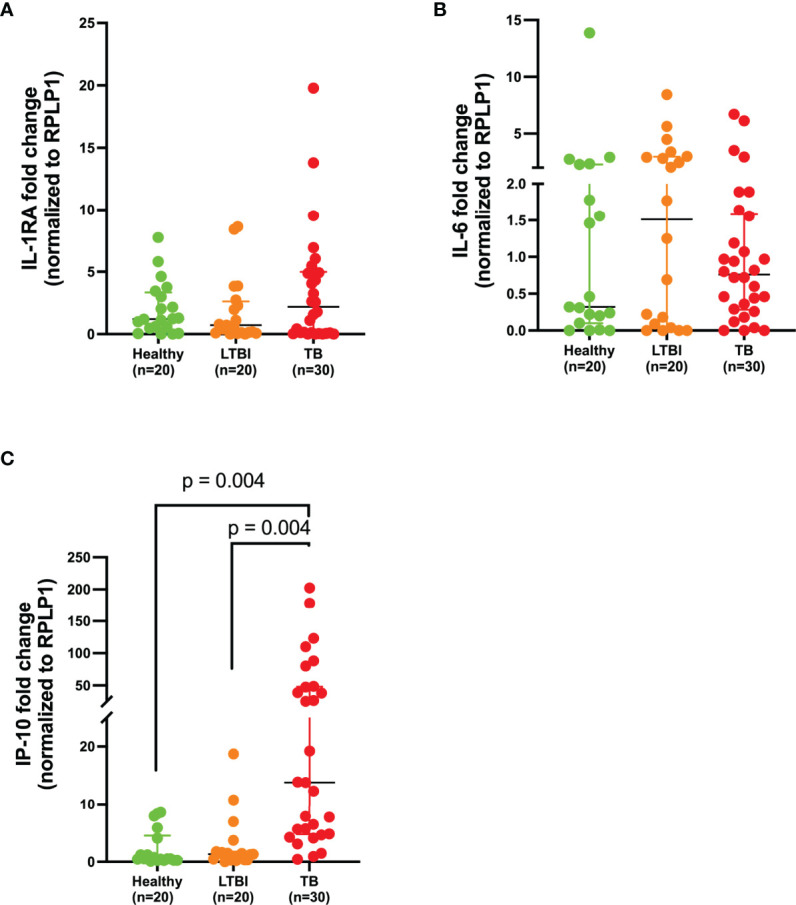
IP-10 is upregulated in whole blood of TB patients. Gene expression of **(A)** IL-1RA, **(B)** IL-6, and **(C)** IP-10 in the whole blood of healthy (n=20), LTBI (n=20), and TB (n=30) participants normalized to a house-keeping gene, RPLP1. *P*< 0.05 was considered statistically significant. Data were analyzed using the one-way ANOVA and followed by a multiple comparisons test using Tukey’s test. Tukey’s *p*-values have been reported in the above figure and illustrate the differences observed between groups.

To determine if the circulatory signatures observed in the plasma were also expressed at the gene level, we measured the expression of IL-1RA, IL-6, and IP-10 in whole blood by qPCR. We found that there was a difference between one of the groups for IP-10 (*p* = 0.001). Tukey’s test found that IP-10 was upregulated in the TB group compared to the LTBI group (*p* = 0.004, 91% CI (-61.37 to -10.44), **Figure 3C**). IP-10 gene was also upregulated in the TB group compared to healthy (*p* = 0.004, 91% CI (-62.33 to -10.55), **Figure 3C**). Our data shows that IP-10 is more abundant at both the protein and gene level, and that it can be explored as a potential biomarker of TB disease. No significant differences were observed for IL-1RA and IL-6 at the mRNA level (**Figures 3A, B**, respectively).

### ROC Analysis Shows That IP-10 and IL-1RA Have Good Discriminatory Power Between Healthy Individuals and TB Patients

**Figure 4:**
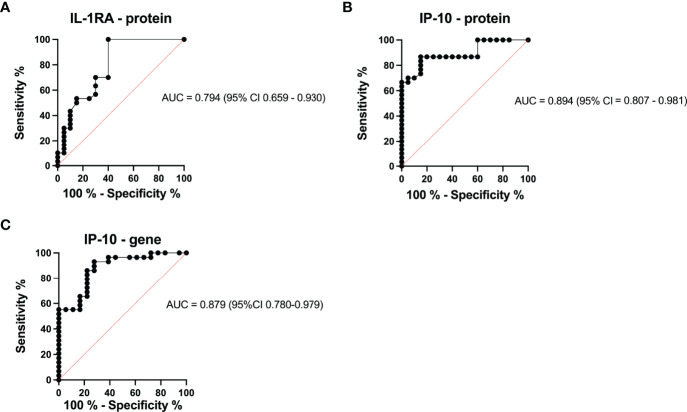
ROC curve characteristics for IP-10 protein and IL-1RA protein as well as IP-10 mRNA, as discriminatory markers for TB. AUC, specificity, sensitivity, PPV, and NPV indicate the power of **(A)** IL-1RA protein abundance, **(B)** IP-10 protein abundance, and **(C)** IP-10 mRNA expression which are reported and indicate the power of these biomarkers for discriminating between those who are healthy and those with TB.

To assess the power of IL-1RA and IP-10 to discriminate between healthy individuals and those with TB, we performed ROC analysis. IL-1RA and IP-10 both showed a good area under the curve (AUC=0.794 and AUC=0.894, **Figures 4A, B**, respectively) with high specificity and sensitivity. IL-1RA had a positive predictive value (PPV) of 86.47% and a negative predictive value (NPV) of 44.95%. IP-10 showed more potential with PPV=92.39% and NPV= 68.15% (**Figure 4B**). To determine if IP-10 could distinguish between healthy and TB patients at the mRNA level, we performed ROC analysis based on the qPCR data. Even at the mRNA level, IP-10 had good positive and negative predictive values (AUC=0.879, with PPV=91, 58% and NPV=66, 79%) with high specificity (86, 20%) and sensitivity (77, 78%) (**Figure 4C**).

## Discussion

This study investigated the potential of using plasma samples to identify circulatory markers that may distinguish between healthy, LTBI, and active TB disease. Of the 27 cytokines/chemokines assessed, IL-1RA, IL-6, and IP-10 were significantly different between individuals with LTBI and TB patients at the plasma protein levels. Gene expression profiles also validated IP-10 as a potential biomarker that can distinguish active TB disease from LTBI at the mRNA level.

A previous study identified the prognostic value of host soluble mediators as biomarkers for TB-associated mortality (Schutz et al., 2019). Common markers reported in our study and that of Schutz et al., identified cytokines IL-1RA, IL-6, and the chemokine IP-10 to be a marker of disease severity (Schutz et al., 2019). Furthermore, other studies have reported that IFN-γ, IL-2, IL-5, IL-10, IL-1RA, and MCP-1 distinguished between individuals with LTBI and TB individuals in QFT supernatants stimulated with TB antigen (Suzukawa et al., 2016). Our study differs from the approach taken by others (Suzukawa et al., 2016) in that we did not stimulate the plasma with any TB antigen. We also determined gene expression levels of the cytokines to validate their capacity to distinguish disease stages at the mRNA level. IP-10 discriminated TB from LTBI participants at both the protein and gene level.

IL-1RA is secreted by monocytes, eosinophils, and neutrophils and it antagonistically binds to IL-1α and IL-1β. Our findings of elevated IL-1RA in the TB group compared to the LTBI group corroborates a previous study which reported increased levels of IL-1RA in the serum of individuals with active TB (Juffermans et al., 1998). In addition, previous studies have also shown elevated levels of IL-1RA in QFT supernatants (Ruhwald et al., 2009) stimulated with TB antigens such as ESAT-6, CFP-10, and TB7.7 (Suzukawa et al., 2016). Given the highly inflammatory nature of TB, the repeated presence of IL-1RA in various settings such as in QFT TB antigen stimulation (Suzukawa et al., 2016), bronchoalveolar lavage (BAL) fluid (Tsao et al., 2000), and its presence in our study, albeit in unstimulated plasma, emphasizes the importance of IL-1RA as a potential biomarker of disease progression. IL-6 is well established as a marker of inflammation and plays a vital role in the cytokine storm associated with TB disease [as reviewed by (Boni et al., 2022)]. The abundant protein levels of IL-6 in the active TB group corroborates with previous findings that reported an association of IL-6 at the plasma level with pulmonary TB disease severity (Zambuzi et al., 2016). IL-6 has also been suggested as a biomarker to monitor TB disease in patients undergoing anti-TB treatment (Singh and Goyal, 2013; Chowdhury et al., 2014).

IP-10 is a chemokine that recruits T-lymphocyte cells to areas of inflammation and regulates the recruitment of Th1 cells (Azzurri et al., 2005; Blauenfeldt et al., 2018). As an interferon gamma inducible protein, the association of IP-10 with active disease supports the known relationship between IFN- γ and TB disease (Kannabus, 2021). Various studies have reported on the potential of IP-10 as a diagnostic marker to discriminate between individuals with LTBI and TB (Jacobs et al., 2016; Kumar et al., 2021) and in patients’ whole blood, stimulated with ESAT-6 and CFP-10 (Borgström et al., 2012; Jeong et al., 2015; Comella-Del-Barrio et al., 2019). IP-10 is highly detectable in the plasma of patients with active TB (Borgström et al., 2012; Jeong et al., 2015), measurably reduced in those on treatment, present in granulomas (Ferrero et al., 2003), and detectable in individuals cohabiting with a TB positive person, including children (Azzurri et al., 2005; Whittaker et al., 2008). Finally, IP-10 secretion is not influenced by the age of the person infected and is therefore useful in diagnosing both adults and children (Whittaker et al., 2008).

We hypothesize that these markers may serve as a good indicator to track and distinguish states in individuals diagnosed with LTBI. Accurate diagnosis may aid in preventative treatment which may reduce the likelihood of individuals progressing to TB disease if diagnosed early.

Our study shows that differences in circulatory cytokines/chemokines exist between individuals with LTBI and TB. These proteins may be exploited as biomarkers of disease progression. We were not able to correlate our circulatory markers with sputum bacterial loads, which would further inform on the relationship between diagnostic markers and bacterial load. This would provide more information on the strength of the immune response produced as a consequence of bacterial burden. However, a strength of our study is the association of the biomarkers with the disease both in the plasma and validation at the mRNA level. In addition, two of the markers we report here, namely IP-10 and IL-6, have also been reported in unstimulated plasma (Yao et al., 2017), as biomarkers for disease progression and validated in larger cohorts (Kumar et al., 2021). In addition, the high specificity and sensitivity observed in our ROC analysis for IP-10 indicate its potential in serving as a good diagnostic marker in conjunction with other inflammatory cytokine signatures. This data needs to be validated in a larger cohort in order to corroborate with previously reported discovery and validation cohorts (Yao et al., 2017; Kumar et al., 2021). The use of several cytokines as diagnostic markers has been suggested, and validating the identified cytokines is crucial for overcoming diagnostic challenges to improve health care guidelines currently in place for treatment of TB.

Our study supports the growing body of evidence which advocates for the use of plasma diagnostic biomarkers such as those detected in the circulation of individuals with LTBI and TB as a potential diagnostic tool. Further studies are needed to investigate the validity and viability of using specific cytokines and chemokines such as IL-1RA, IL-6, and IP-10 as potential biomarkers of disease progression in a high-disease burden setting.

## Data Availability Statement

The original contributions presented in the study are included in the article/supplementary material. Further inquiries can be directed to the corresponding author.

## Ethics Statement

The studies involving human participants were reviewed and approved by the Biomedical Research Ethics Committee (BREC) at the University of KwaZulu-Natal (BE022/13). The patients/participants provided their written informed consent to participate in this study.

## Author Contributions

KF performed experimental work and wrote the manuscript. KF and MJM performed data analysis. OB, DM, and KR-B supported with experimental work and sample preparation. FK provided clinical samples. MJM, HN, and TN conceptualized the project and obtained funding for this study. All authors contributed to the article and approved the submitted version.

## Funding

The work reported herein was made possible through funding by the South African Medical Research Council (SAMRC) through its Division of Research Capacity Development under the Internship Scholarship Programme (KF) and SAMRC Mid-Career Scientist Programme (MM) with funding received from the South African National Treasury. The content hereof is the sole responsibility of the authors and does not necessarily represent the official views of the SAMRC or the funders. MM was funded by the Wellcome Trust (grant#206751/A/17/Z) and Grand Challenges, an initiative of the Bill & Melinda Gates Foundation (grant #OPP1210776, grant# INV-016239), and SAMRC with funding from the SA Department of Health. HN was funded by NRF CSUR (grant # 116260).

## Conflict of Interest

The authors declare that the research was conducted in the absence of any commercial or financial relationships that could be construed as a potential conflict of interest.

## Publisher’s Note

All claims expressed in this article are solely those of the authors and do not necessarily represent those of their affiliated organizations, or those of the publisher, the editors and the reviewers. Any product that may be evaluated in this article, or claim that may be made by its manufacturer, is not guaranteed or endorsed by the publisher.
